# Effects of overinflation on procollagen type III expression in experimental acute lung injury

**DOI:** 10.1186/cc5702

**Published:** 2007-02-21

**Authors:** Maria-Eudóxia Pilotto de Carvalho, Marisa Dolhnikoff, Sibele Inácio Meireles, Luiz Fernando Lima Reis, Milton Arruda Martins, Daniel Deheinzelin

**Affiliations:** 1 Intensive Care Unit, Centro de Tratamento e Pesquisa, Hospital do Câncer, Fundação Antônio Prudente; Rua Prof. Antônio Prudente, 211; São Paulo; CEP: 01509-010; Brazil; 2 Department of Pathology, School of Medicine, University of São Paulo; Avenida Dr. Arnaldo, 455; São Paulo; CEP: 01246-000; Brazil; 3 Ludwig Institute of Cancer Research, Centro de Tratamento e Pesquisa, Hospital do Câncer; Rua Prof. Antônio Prudente, 211; São Paulo; CEP: 01509-010; Brazil; 4 Laboratório de Investigação Médica 20, School of Medicine, University of São Paulo; Avenida Dr. Arnaldo, 455; São Paulo; CEP: 01246-000; Brazil

## Abstract

**Introduction:**

In acute lung injury (ALI), elevation of procollagen type III (PC III) occurs early and has an adverse impact on outcome. We examined whether different high-inflation strategies of mechanical ventilation (MV) in oleic acid (OA) ALI alter regional expression of PC III.

**Methods:**

We designed an experimental, randomized, and controlled protocol in which rats were allocated to two control groups (no injury, recruited [alveolar recruitment maneuver after tracheotomy without MV; *n *= 4 rats] and control [*n *= 5 rats]) or four injured groups (one exposed to OA only [*n *= 10 rats] and three OA-injured and ventilated). The three OA-injured groups were ventilated for 1 hour according to the following strategies: LVHP-S (low volume-high positive end-expiratory pressure [PEEP], supine; *n *= 10 rats, tidal volume [V_T_] = 8 ml/kg, PEEP = 12 cm H_2_O), HVLP-S (high volume-low PEEP, supine; *n *= 10 rats, V_T _= 20 ml/kg, PEEP = 5 cm H_2_O), and HVLP-P (high volume-low PEEP, prone; *n *= 10 rats). Northern blot analysis for PC III and interleukin-1-beta (IL-1β) and polymorphonuclear infiltration index (PMI) counting were performed in nondependent and dependent regions. Regional differences between groups were assessed by two-way analysis of variance after logarithmic transformation and *post hoc *tests.

**Results:**

A significant interaction for group and region effects was observed for PC III (*p *= 0.012) with higher expression in the nondependent region for HVLP-S and LVHP-S, intermediate for OA and HVLP-P, and lower for control (group effect, *p *< 0.00001, partial η^2 ^= 0.767; region effect, *p *= 0.0007, partial η^2 ^= 0.091). We found high expression of IL-1β (group effect, *p *< 0.00001, partial η^2 ^= 0.944) in the OA, HVLP-S, and HVLP-P groups without regional differences (*p *= 0.16). PMI behaved similarly (group effect, *p *< 0.00001, partial η^2 ^= 0.832).

**Conclusion:**

PC III expression is higher in nondependent regions and in ventilatory strategies that caused overdistension. This response was partially attenuated by prone positioning.

## Introduction

Over the past decades, mechanical ventilation (MV) has been employed as the main supportive tool in the setting of severe respiratory failure. Lung parenchyma and in particular extracellular matrix (ECM) are exposed to physical stimuli during MV, which may produce an adaptive response. ECM is composed of water and biological macromolecules such as collagens, elastin, and proteoglycans [1], of which collagens are the most abundant and are responsible for structural integrity [2]. Our knowledge of the consequences of MV in the ECM of normal [3] and diseased [4] lungs has expanded recently. Injurious MV subjects lung parenchyma to high inflation and initiates ECM remodeling in patients [5] and experimental models [6-9]. This event depends on an airway pressure (P_AW_) gradient [6,7] and a transpleural pressure gradient. In fact, in healthy rat lungs submitted to injurious ventilation either with high or low tidal volume (V_T_) values, ECM reacted with an increased synthesis of mRNA for procollagen type III (PC III), which was more pronounced in nondependent regions of the lungs [10]. This suggests an effect of regional transpleural forces that emerged due to lung heterogeneity in the context of ventilator-induced lung injury [11].

On the other hand, pulmonary fibrosis is a consequence of acute lung injury (ALI) and contributes to prolonged respiratory failure and ultimately death in acute respiratory distress syndrome (ARDS) [12,13]. Excessive collagen synthesis is an important part of this biological response [14]. Moreover, different approaches have shown that early elevation of PC III is a predictor of poor outcome in patients with ARDS [15-18].

We investigated how the initial fibroproliferative adaptive response interacts with MV of injured lungs. Because regional forces influence the fibroproliferative response [10], we employed different high-inflation ventilatory strategies to observe how they would affect the transcription of PC III mRNA in nondependent and dependent regions of rat lungs exposed to oleic acid (OA) and ventilated for one hour. High and low positive end-expiratory pressure (PEEP) levels were used to obtain similar degrees of high peak P_AW _values with different cyclic stretch. We also studied animals that were in the prone position, which is known to reduce the transpleural pressure gradient [19]. mRNA expression was chosen due to short experiment length. Steady-state synthesis of procollagen type I (PC I) can be affected by alterations in messenger stability [20,21] or in transcriptional rate [20]. Nevertheless, studies have shown that an increase in mRNA PC I is consistent with an increase in PC I protein levels [22-24]. The same has been verified for PC III [25].

To confirm the degree of lung injury in this early-phase model of ALI [26], we measured mRNA expression of interleukin-1-beta (IL-1β), which is a net mediator of inflammatory activity [27] in addition to being secreted early in the process [10,28] and responsive to changes in ventilatory strategies [29]. Also, we histologically verified the intensity of the polymorphonuclear infiltrate using a polymorphonuclear infiltrarion index (PMI).

## Materials and methods

The study was approved by the Ethics Committee on Clinical Research and the Ethics Committee for Animal Experimentation of the Hospital do Câncer (São Paulo, Brazil). Animals were treated according to internal standards for animal experimentation.

We studied six groups of male Wistar rats. After anesthesia (ketamine 80 mg/kg and xylazine 10 mg/kg), tracheotomy, jugular vein and carotid artery sections, rats were placed in the prone position and given a slow intravenous bolus of 30 μl of OA (Sigma-Aldrich, St. Louis, MO, USA) dissolved in 270 μl of bovine serum albumin. After stabilization (15 minutes), three groups of 10 randomly assigned rats were ventilated for one hour in a volume-controlled ventilator (Inter-3; Intermed Equipamento Médico Hospitalar LTDA, São Paulo, Brazil) according to the following strategies to achieve the same peak inspiratory pressure:

1. LVHP-S (low volume-high PEEP, supine): V_T _= 8 ml/kg and PEEP = 12 cm H_2_O in the supine position.

2. HVLP-S (high volume-low PEEP, supine): V_T _= 20 ml/kg and PEEP = 5 cm H_2_O in the supine position.

3. HVLP-P (high volume-low PEEP, prone): V_T _= 20 ml/kg and PEEP = 5 cm H_2_O in the prone position. Thoracic and pelvic cushions were placed to free the abdominal wall.

### Mechanical ventilation

Briefly, rats were connected to a small animal micro-processor ventilator (Inter-3; Intermed) in series with a pneumotachograph (8420; Hans Rudolph, Inc., Kansas City, MO, USA). Flow V' and tracheal pressure P_AW _were measured by a differential pressure transducer (DP45-16-2114; Validyne Engineering, Northridge, CA, USA) and a pressure transducer (DP45-28-2114; Validyne). These signals were amplified (RS 3400; Gould Electronics, Inc., Chandler, AZ, USA) and converted (DT 2801; Data Translation, Inc., Marlboro, MA, USA). Further digital processing with PC software ANADAT 4.0/LABDAT 4.0 (RHT-Info Dat, Montreal, Canada) produced records of P_AW_, V', and volume V (time integral of V'). For all ventilatory strategies, fraction of inspired oxygen (FiO_2_) was 40% and respiratory rate was kept at 90 breaths per minute.

Three other groups were not ventilated:

1. OA: Ten OA-injected rats breathed spontaneously for one hour in the supine position, and the degree of lung injury without the effects of MV was assessed.

In the other two groups, baseline morphometry and mRNA expression were studied:

2. No injury, recruited (NIR): To assess morphometry, after anesthesia and tracheotomy, four rats were recruited with continuous positive airway pressure of 30 cm H_2_O for 30 seconds to overcome atelectasis formation due to anesthesia. [30].

3. Control (C): Five rats were sacrificed after anesthesia for RNA studies since even isolated parenchymal distensions that occur during a recruitment maneuver may lead to increased procollagen expression. [8].

Mean arterial pressure was monitored, and saline was infused through the venous line to keep it above 60 mm Hg. Arterial blood gases were performed before sacrifice in the three ventilated groups.

Animals were then bled to death and their lungs and heart were harvested *en bloc *after tracheal occlusion to maintain a static inflation pressure of 5 cm H_2_O. Approximately 1 cm^3 ^of tissue was obtained from nondependent (sternal edge) and dependent (caudal and dorsal) portions of the left lung, avoiding central areas of large bronchi and vessels, and was frozen for mRNA analysis. Nondependent (the medium lobe) and dependent (caudal and dorsal area of the inferior lobe) portions of the right lung were obtained after formalin fixation, and a 2-μm-thick slide from each portion was stained with hematoxylin-eosin for morphometry.

Using the point-counting method [31] and a 100-point grid attached to the ocular of the microscope, the PMI was estimated as the ratio of the number of points that fell on polymorphonuclear (PMN) cells to the number of points that fell on the alveolar septum. Counting was carried out in 15 randomly chosen fields per slide, at a × 400 magnification, by two investigators who were blinded to the case and region of sampling. The coefficient of variation for the interobserver error for cell counts was less than 5%. Data were expressed as the logarithm of PMI (as logPMI).

IL-1β and PC III mRNA expressions were determined by Northern blot analysis using total RNA [32], the probes previously described [10], and glyceraldehyde-3-phosphate dehydrogenase (GAPDH) as control for RNA loading. Filters were scanned by a phosphorimager (Storm 840; Molecular Dynamics, now part of GE Healthcare, Little Chalfont, Buckinghamshire, UK). Data were expressed as the logarithm of the probe/GAPDH ratio (as logIL1 and logpcIII).

Control variables were not normally distributed and were described by median and interquartile ranges and compared by Kruskal-Wallis or Mann-Whitney *U *tests when appropriate. mRNA expressions of PC III and IL-1β and PMI were reported as their logarithmic functions and described as means and standard deviations. Regional differences between groups in mRNA expression and PMI were assessed by two-way analysis of variance (ANOVA) for repeated measures after the logarithmic transformation to ensure normality of distributions and homogeneity of variances (verified by Kolmogorov-Smirnov and Levene tests, respectively). *Post hoc *analysis was then performed (Tuckey honest significant difference). For all tests, α = 0.05. Statistical analysis was performed with SPSS 13.0 software (SPSS Inc., Chicago, IL, USA).

## Results

The animals were similar in regard to weight (all groups), doses of anesthetic agents and volume of saline infused (for LVHP-S, HVLP-S, HVLP-P, and OA groups), P_AW _(for the ventilated groups LVHP-S, HVLP-S, and HVLP-P), and V_T _and PEEP (for HVLP-P and HVLP-S). Results (medians and interquartile ranges) are shown in Table 1.

**Table 1:** Comparison of control and ventilatory variables

	LVHP-S	HVLP-S	HVLP-P	OA	NIR	C	*p*
	*n *= 10	*n *= 10	*n *= 10	*n *= 10	*n *= 4	*n *= 5	
	Median (25–75)	Median (25–75)	Median (25–75)	Median (25–75)	Median (25–75)	Median (25–75)	
Weight (kg)	0.22 (0.025–0.23)	0.26 (0.23–0.27)	0.255 (0.23–0.28)	0.245 (0.225–0.26)	0.263 (0.22–0.293)	0.255 (0.24–0.26)	0.35
Saline (ml/kg)	2.63 (2.2–3.3)	2.3 (1.55–3.3)	1.8 (1.1–4.0)	1.43 (0.9–2.4)	--	--	0.20^a^
V_T_(ml/kg)	8.8 (8.5–10.1)	18.2 (17.2–20.6)	18.59 (13.5–24.6)	--	--	--	0.82^b^
PEEP (cm H_2_O)	12.13 (12.03–12.16)	5.27 (5.09–5.46)	5.26 (5.07–5.39)	--	--	--	0.85^b^
P_AW _(cm H_2_O)	24.97 (23.57–25.59)	23.7 (22.94–23.86)	23.22 (22.99–23.56)	--	--	--	0.09^c^
pH	7.05 (7.05–7.10)	7.51 7.46–7.55)	7.50 (7.47–7.54)	--	--	--	--
pO_2 _(mmHg)	178.9 (169.6–193.9)	177.0 (131-8–226.4)	158.4 (115.3–188.0)	--	--	--	--
pCO_2 _(mmHg)	70.7 (64.2–90.1)	11.9 (11.5–13.1)	14.1 (12.0–17.3)	--	--	--	--

Results are expressed as median and interquartile range (25–75). ^a^Kruskal-Wallis test between LVHP-S, HVLP-S, HVLP-P, and OA; ^b^Mann-Whitney test between HVLP-S and HVLP-P; ^c^Kruskal-Wallis test between LVHP-S, HVLP-S, and HVLP-P. C, control group; HVLP-P, high volume-low positive end-expiratory pressure, prone; HVLP-S, high volume-low positive end-expiratory pressure, supine; LVHP-S, low volume-high positive end-expiratory pressure, supine; NIR, no injury, recruited; OA, oleic acid injury, no ventilation; P_AW_, peak airway pressure; pCO_2_, carbon dioxide partial pressure; PEEP, positive end-expiratory pressure; pO_2_, oxygen partial pressure; V_T_, tidal volume.

The administration of OA effectively induced lung injury and resulted in a decrease in pO_2_/FiO_2 _ratio, perivascular and alveolar septa edema, and (as expected) marked PMN infiltration [33]. The groups ventilated with high V_T _(HVLP-S and HVLP-P) presented marked alkalosis due to low carbon dioxide partial pressure (pCO_2_). Conversely, the low-V_T _LVHP-S group showed acidosis due to high pCO_2 _at the end of the experiment.

**Figure 1:**
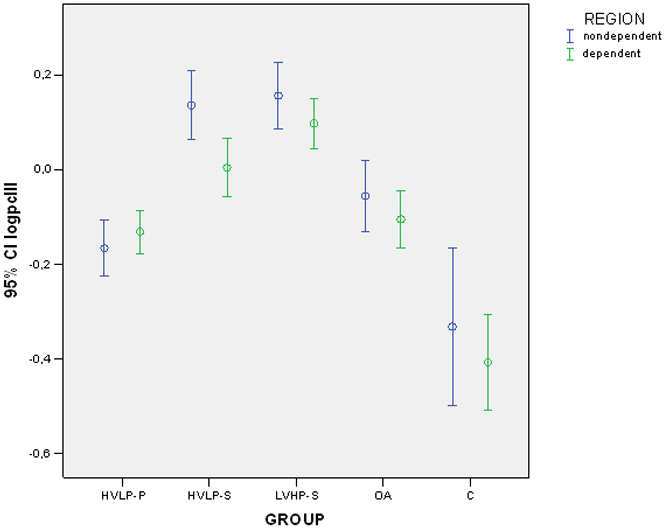
Logarithm of the relative expression of procollagen type III mRNA and GAPDH obtained by Northern blotting in the nondependent and dependent regions of the left lung. Error bars represent mean and 95% confidence interval (CI). C, control group; GAPDH, glyceraldehyde-3-phosphate dehydrogenase; HVLP-P, high volume-low positive end-expiratory pressure, prone; HVLP-S, high volume-low positive end-expiratory pressure, supine; logpcIII: logarithmic transformation of the expression of PC III mRNA normalized by GAPDH mRNA; LVHP-S, low volume-high positive end-expiratory pressure, supine; OA, oleic acid injury, no ventilation.

Expression of PC III for each group and region is shown in Figure 1. A significant interaction for group and region effects was observed for the expression of PC III (for the interaction *p *= 0.012, ANOVA two-way) with higher expression in the HVLP-S and LVHP-S groups (group effect, *p *< 0.00001, ANOVA two-way, partial η^2 ^= 0.767) and in the nondependent region (region effect, *p *= 0.0007, ANOVA two-way, partial η^2 ^= 0.091). *Post hoc *analysis showed that the expression of PC III was high in the HVLP-S and LVHP-S groups, intermediate in the OA and HVLP-P groups, and low in the control group. The expression of PC III was higher in the nondependent region of the LVHP-S and HVLP-S groups compared to the dependent region of the HVLP-S group. Results (means and standard deviations) and significant differences between groups or regions after *post hoc *analysis are shown in Table 2.

**Table 2:** Procollagen type III mRNA expression (logpcIII) sorted by lung region

	LVHP-S	HVLP-S	HVLP-P	OA	NIR	C
	*n *= 10	*n *= 10	*n *= 10	*n *= 10	*n *= 4	*n *= 5

	Mean (SD)	Mean (SD)	Mean (SD)	Mean (SD)	Mean (SD)	Mean (SD)

logpcIII nondependent	0.1564 (0.0971)^a^	0.136 (0.1009)^a^	-0.1657 (0.0826)	-0.552 (0.1042)	--	-0.3314 (0.1345)
logpcIII dependent	0.0978 (0.0744)	0.0045 (0.085)^a^	-0.1309 (0.0636)	-0.1047 (0.0852)	--	-0.4067 (0.0812)

Results are expressed as mean and standard deviation (SD). *Post hoc *analysis: (1) significant differences found within groups in nondependent versus dependent regions: ^a^HVLP-S, *p *= 0.004; LVHP-S nondependent versus HVLP-S dependent, *p *= 0.0007; (2) significant differences found between groups: (i) HVLP-P versus HVLP-S, HVLP-P versus LVHP-S, and HVLP-P versus C, *p *< 0.001; (ii) HVLP-S versus OA and HVLP-S versus C, *p *< 0.001; (iii) LVHP-S versus OA and LVHP-S versus C, *p *< 0.001; (iv) OA versus C, *p *< 0.001. C, control group; HVLP-P, high volume-low positive end-expiratory pressure, prone; HVLP-S, high volume-low positive end-expiratory pressure, supine; logpcIII: logarithmic transformation of the expression of PC III mRNA normalized by GAPDH mRNA; LVHP-S, low volume-high positive end-expiratory pressure, supine; NIR, no injury, recruited; OA, oleic acid injury, no ventilation.

**Figure 2:**
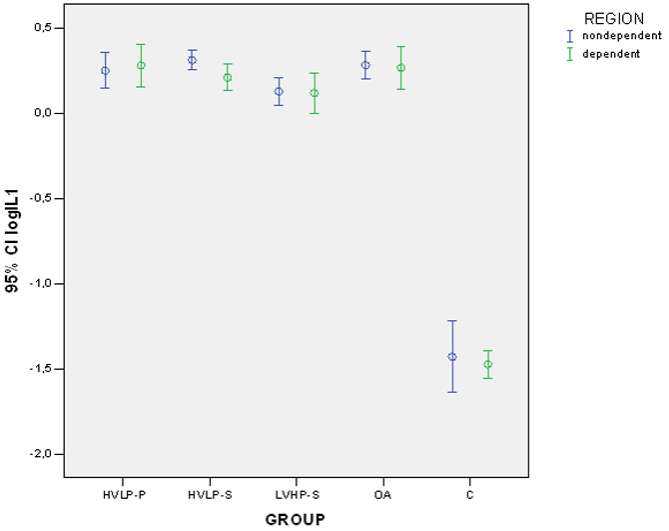
Logarithm of the relative expression of interleukin-1-beta mRNA and GAPDH obtained by Northern blotting in the nondependent and the dependent regions of the left lung. Error bars represent mean and 95% confidence interval (CI). C, control group; GAPDH, glyceraldehyde-3-phosphate dehydrogenase; HVLP-P, high volume-low positive end-expiratory pressure, prone; HVLP-S, high volume-low positive end-expiratory pressure, supine; logIL1: logarithmic transformation of the expression of IL-1β mRNA normalized by GAPDH mRNA; LVHP-S, low volume-high positive end-expiratory pressure, supine; OA, oleic acid injury, no ventilation.

**Figure 3:**
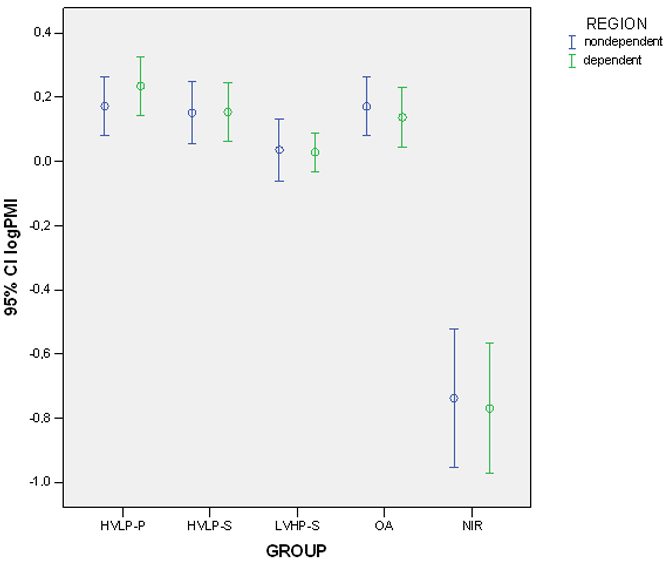
Logarithm of the polymorphonuclear infiltration index in the nondependent and dependent regions of the right lung. Error bars represent mean and 95% confidence interval (CI). C, control group; HVLP-P, high volume-low positive end-expiratory pressure, prone; HVLP-S, high volume-low positive end-expiratory pressure, supine; logPMI, logarithm of the polymorphonuclear infiltration index; LVHP-S, low volume-high positive end-expiratory pressure, supine; NIR, no injury, recruited; OA, oleic acid injury, no ventilation.

Expression of IL-1β and PMI sorted by group and region are shown in Figures 2 and 3. Variables exhibited similar behavior. There was a significant group effect on the expression of IL-1β (group effect, *p *< 0.00001, ANOVA two-way, partial η^2 ^= 0.944) without regional differences (region effect, *p *= 0.16, ANOVA two-way, partial η^2 ^= 0.011). *Post hoc *analysis confirmed that there was minimal (control), intermediate (LVHP-S), and high (HVLP-S, HVLP-P, and OA) expression of IL-1β. Results (means and standard deviations) of IL-1β and PMI followed by significant differences between groups after *post hoc *analysis are shown in Table 3.

**Table 3:** IL-1β mRNA expression (logIL1) and PMI (logPMI) sorted by lung region

	LVHP-S	HVLP-S	HVLP-P	OA	NIR	C
	*n *= 10	*n *= 10	*n *= 10	*n *= 10	*n *= 4	*n *= 5

	Mean (SD)	Mean (SD)	Mean (SD)	Mean (SD)	Mean (SD)	Mean (SD)

logIL1 nondependent	0.1262 (0.1117)	0.3102 (0.0807)	0.2479 (0.1472)	0.2804 (0.1129)	--	-1.4306 (0.1692)
logIL1 dependent	0.1166 (0.1674)	0.2078 (0.1079)	0.2784 (0.1748)	0.2657 (0.1758)	--	-1.4749 (0.0646)
logPMI nondependent	0.0363 (0.1349)	0.1518 (0.1345)	0.1725 (0.1255)	0.1721 (0.1273)	-0.738 (0.1359)	--
logPMI dependent	0.0291 (0.0848)	0.1545 (0.1286)	0.2347 (0.1272)	0.1372 (0.1287)	-0.7697 (0.1276)	--

Results are expressed as mean and standard deviation (SD). *Post hoc *analysis for IL-1β expression: significant differences found between groups: (i) C versus HVLP-P, C versus HVLP-S, C versus LVHP-S, and C versus OA, *p *< 0.001; (ii) LVHP-S versus HVLP-P, *p *= 0.014; (iii) LVHP-S versus HVLP-S, *p *= 0.019; (iv) LVHP-S versus OA, *p *= 0.007. *Post hoc *analysis for PMI: significant differences found between groups: (i) NIR versus HVLP-P, NIR versus HVLP-S, NIR versus LVHP-S, and NIR versus OA, *p *< 0.001; (ii) LVHP-S versus HVLP-P, *p *< 0.001; (iii) LVHP-S versus HVLP-S, *p *= 0.026; (iv) LVHP-S versus OA, *p *= 0.024. C, control group; HVLP-P, high volume-low positive end-expiratory pressure, prone; HVLP-S, high volume-low positive end-expiratory pressure, supine; IL-1β, interleukin-1-beta; logIL1: logarithmic transformation of the expression of IL-1β mRNA normalized by GAPDH mRNA; logPMI, logarithm of the polymorphonuclear infiltratrion index; LVHP-S, low volume-high positive end-expiratory pressure, supine; NIR, no injury, recruited; OA, oleic acid injury, no ventilation; PMI, polymorphonuclear infiltrate.

We noted a very low PMN infiltration as characterized by logPMI in the NIR group, an intermediate degree of infiltration in the LVHP-S group, and a high level of infiltration in the HVLP-S, HVLP-P, and OA groups (group effect, *p *< 0.00001, ANOVA two-way, partial η^2 ^= 0.832) as confirmed by *post hoc *analysis. No regional differences were observed (region effect, *p *= 0.9, ANOVA two-way, partial η^2 ^< 0.001).

## Discussion

Our main findings were the following: First, upregulation of PC III expression occurred early in this ALI model; second, it was significantly higher in ventilatory strategies that possibly generated overinflation due to the fact that either high PEEP or high V_T _affected mostly nondependent lung regions of these groups; and third, the prone position partially attenuated this response.

The early response of PC III mRNA is in accordance with previous studies [5] that have shown that mRNA expression of PC I increases very early in the course of extracorporeal circulation for cardiopulmonary bypass surgery. Injuriously high V_T _ventilation is also capable of rapidly inducing transforming growth factor-beta-1 mRNA, an upstream regulator of collagen synthesis [34]. In experimental models, increased alveolar wall stress during a four hour period was accompanied by an increased synthesis of PC I, PC III, PC IV, and laminin B [6]. Besides, it is known that prolonged alveolar distension of the remaining lung after pneumonectomy causes an increased transcription of collagen [22,35]. Taken together, these findings suggest that overdistension due to MV leads to an early response of the ECM.

Moreover, we found significantly higher expression of PC III mRNA with an effect size of 77% in ventilatory strategies associated with overinflation of lung parenchyma, as we noticed in the HVLP-S and LVHP-S groups, regardless of how high end-inspiratory volume was achieved. Additionally, nondependent regions of the latter groups were particularly exposed to the accumulation of PC III mRNA, although this effect was somewhat less (9%). Considering the OA model, the use of strategies characterized by high V_T _or high PEEP may lead to higher end inspiratory lung volume in nondependent regions [36], rendering them more susceptible to mechanical strain. Accordingly, there is indirect evidence of regional overinflation in human studies. Treggiari and colleagues [37] observed more cystic lesions in the nondependent lung regions (middle lobe and anterior and medial basal segments of the lower lobe) of patients in the fibroproliferative phase of ARDS, thus suggesting a potential mechanism for triggering PC III mRNA response.

We observed that rat lungs ventilated in the prone position showed less upregulation for the expression of PC III as compared to MV with high V_T _(HVLP-S) or high PEEP (LVHP-S) for the same peak inspiratory pressure. Indeed, levels of PC III found in the prone group were similar to the unventilated OA group. Prone positioning is associated with increased stiffness of the thoracic cage [38]. Besides, lung inflation [39] and regional gas [40] are more evenly distributed than in the supine position, contributing to a more homogenous distribution of strain throughout lung parenchyma. Much has been learned of the pleural inflation gradient from studies with humans and larger animals [38-40], but to extend this knowledge to a smaller animal like the rat merits concern. Nevertheless, Negrini and coworkers [41] unequivocally demonstrated an increasing transpleural pressure from top (sternum) to bottom (vertebra) in supine rats. In addition, the distribution of lung inflation is more homogeneous in rats in the prone position as compared to the supine position, as shown by computed tomography [19]. This might reduce the overdistension observed in nondependent areas in the supine position, thus preventing an excessive activation of PC III mRNA synthesis.

Although we chose only one cytokine (IL-1β), which might limit the examination of the inflammatory response in relation to fibrogenesis [42], and a semiquantitative histological index (PMI), our findings are in agreement with other experimental studies [43-45]. mRNA expression of IL-1β paralleled the PMI index. We saw a marked expression/infiltration in the OA, HVLP-P, and HVLP-S groups. The LVHP-S group had an intermediate expression/infiltration compared to the high-V_T _strategies and to the injured but not ventilated OA group. Studies that employed strategies of low V_T _(6 to 8 ml/kg) combined with higher PEEP obtained lower levels of proinflammatory cytokines both in humans [46] and animals [43] as opposed to high levels of inflammatory cytokines [44] or high expression of cytokine mRNA [45] observed with high-V_T _ventilation in animal studies. Interestingly, in the present study, this protective effect was detected early in the course of lung injury.

Due to study design, we observed hypercapnia in the LVHP-S group (mean pCO_2 _= 63.5 mm Hg, 95% confidence interval [CI] = 46.5 to 73.4) whereas hypocapnia was noticed in the two high-V_T _groups (for HVLP-S: mean pCO_2 _= 24.2 mm Hg, 95% CI = 13.8 to 36.2; for HVLP-P: mean pCO_2 _= 23.5 mm Hg, 95% CI = 20.2 to 48.9). Could CO_2 _and pH fluctuations influence the inflammatory response observed in our model? It is known that hypercapnia *per se *and hypocapnia have opposite effects in the development of lung injury. Several laboratory studies have suggested that, due to a variety of mechanisms, hypercapnia could be protective in the setting of ALI. These mechanisms (reviewed at length elsewhere [47]) include enhanced anti-inflammatory effects (diminished levels of cytokines, altered neutrophil cell wall adhesion, and reduced lung neutrophil recruitment), lowered free radical species generation and tissue-induced damaged, attenuation of pulmonary apoptosis, and regulation of gene expression (modifying the activation of the transcription factor nuclear factor-kappa B [NF-κB] and differential microarray gene expression [48]). In contrast, hypocapnia presents potential risks of increasing lung injury [49]. This might help explain the differences in the expression of IL-1β and PMI between the low-V_T _hypercapnic LVHP-S group and the two high-V_T _hypocapnic groups.

If there is fairly consistent literature on the effects of hyper/hypocapnia on lung injury, the same is not true for lung repair, particularly collagen synthesis. The effects of acidosis/alkalosis on lung ECM protein synthesis are largely unknown. For that matter, metabolic acidosis induced a decrease in mRNA PC I synthesis in cultured mouse osteoblasts [50], but respiratory acidosis due to hypercapnia did not [51]. Even supposing that hyper/hypocapnia could alter the expression of PC III, we could assume that in our model these effects were marginal in view of the effects of ventilatory strategy; one hypercapnic group (LVHP-S) and a hypocapnic group (HVLP-S) shared high expressions of PC III and the other hypocapnic group (HVLP-P) had significantly less expression of it.

We did not notice significant regional differences in the expression of IL-1β and the PMI. This is in accordance with two recent studies with small animals (rat and rabbit) [19,52], which failed to demonstrate regional differences in the morphology of lung injury in either of the body positions through semiquantitative or subjective evaluation, respectively. However, data from larger animals such as dog and sheep suggested less edema formation and a lower histological injury score in the prone position as compared to the supine position [53,54]. This divergence could be attributed to species size and to methodological differences in the histological parameters chosen (point counting in our study as opposed to scores).

## Conclusion

Our data suggest that in injured lungs ventilation strategy not only may alter the overall procollagen response but also induces a regional fibrogenic response. In the development of better protective ventilatory strategies, all attempts should be made to avoid regional overdistension, thereby reducing any early stimulus for fibrogenesis, which could potentially have an impact on the outcome of patients with ALI/ARDS.

## Key messages

• Upregulation of PC III expression occurred early in this OA ALI model.

• Upregulation of PC III expression was significantly higher in ventilatory strategies that possibly generated overinflation due to the fact that either high PEEP or high V_T _affected mostly nondependent lung regions of these groups.

• The prone position partially attenuated this response.

## Abbreviations

ALI = acute lung injury; ANOVA = analysis of variance; ARDS = acute respiratory distress syndrome; CI = confidence interval; ECM = extracellular matrix; FiO_2 _= fraction of inspired oxygen; GAPDH = glyceraldehyde-3-phosphate dehydrogenase; HVLP-P = high volume-low positive end-expiratory pressure, prone; HVLP-S = high volume-low positive end-expiratory pressure, supine; IL-1β = interleukin-1-beta; logPMI = logarithm of the polymorphonuclear infiltrate; LVHP-S = low volume-high positive end-expiratory pressure, supine; MV = mechanical ventilation; NIR = no injury, recruited; OA = oleic acid; P_AW _= airway pressure; PC I = procollagen type I; PC III = procollagen type III; PC IV = procollagen type IV; pCO_2 _= carbon dioxide partial pressure; PEEP = positive end-expiratory pressure; PMI = polymorphonuclear infiltration index; PMN = polymorphonuclear; pO_2_: oxygen partial pressure; V_T _= tidal volume.

## Competing interests

The authors declare that they have no competing interests.

## Authors' contributions

M-EPC carried out the experiments involving MV of the living animals, mRNA extraction, and Northern blotting, performed histomorphometric countings, and drafted the manuscript. SIM supervised all molecular assays. MD performed histomorphometry and helped to draft the manuscript. MAM participated in the study design, particularly assisting in the MV experiments, and helped to draft the manuscript. LFLR participated in study design, particularly in the choice of molecular assays, and helped to draft the manuscript. DD conceived of the study, participated in its design and coordination, and helped to draft the manuscript. All authors read and approved the final manuscript.
